# Supplementary material for: Hidden multistate models to study multimorbidity trajectories
Source: Sci Rep. 2026 Jul 31;16:23711. doi: 10.1038/s41598-026-62471-w (PMC13427825; doi:10.1038/s41598-026-62471-w)
Supplement: Supplementary file 1 — Supplementary Information. [file 41598_2026_62471_MOESM1_ESM.docx]

**The details of the algorithm employed to generate the data are as follows:**

For each dataset *n* (*n* = 1, 2, …, *N*) and for each subject *k* (*k* = 1, 2, …, *Nsim*):

1. Draw covariate 1 from a binomial distribution:
   x_1~ Binomial(*p* = 0.45), where 1 corresponds to female and 0 to male.
2. Draw covariate 2 from a binomial distribution:
   Educ x_2 ~ Binomial(*p* = 0.15), where 1 corresponds to elementary education as the highest level of education and 0 to higher education levels.
3. Draw age at entry from a truncated gamma distribution with shape 0.9 and rate 0.15, restricted to values between 60 and 96:
   A_k ~ Gamma(α = 0.9, β = 0.15), truncated to 60 ≤ A_k ≤ 96.
4. Draw time in the study from a uniform distribution:
   T_k ~ Uniform(0.5, 20).
5. Simulate the cluster at entry and then simulate the diseases at baseline conditioned on the cluster to which each subject belongs. For each disease, draw from a binomial distribution with probability *p* determined by the latent class model (the probability of developing a certain disease given a certain multimorbidity cluster and the age at entry).
6. Simulate latent multimorbidity cluster trajectories from a multi-state model with Gompertz hazard, adjusted for the binary covariates.
7. Simulate the prevalent diseases (for those the patient has not developed yet) conditioned on the latent cluster towards which the patient is transitioning. If the next state of transition is death, diseases are simulated conditioned on the current state.
8. Simulate the age of onset for each developed disease from a truncated beta distribution based on disease-specific parameters. The distribution is truncated so that the age of onset is between the transition from the previous state to the next. If this age exceeds the age of death, the corresponding simulated disease is discarded.
9. Simulate rare diseases independently from the states to which patients belong but dependently on the age of the patient. Rare diseases are drawn from a binomial distribution with parameter *p* equal to the prevalence of such diseases stratified by age (as reported in below).
10. Remove subjects who do not present multimorbidity at baseline.
11. Compute the age of exit from the study as the minimum between the age of death and the age of entry plus time in study.
12. Eliminate data after the age of exit (in the case of patients who leave the study before dying).

From the disease-generating mechanism described above, the underlying "true" datasets (ground truth) are obtained. Subsequently, study design schemes (population study or irregular visits) are applied to transform the data into what would be observed in reality when collecting multimorbidity data. Since the exact time of disease onset is usually unknown in reality, the information about when the diseases are developed for each patient is replaced by binary-coded variables. A value of 1 indicates that the patient has developed the disease before the time *t* of follow-up, and 0 otherwise. As a result, information about disease onset is not present in the final datasets.

In the case of the population study, the times of visits *t* are deterministically simulated starting from the age of entry in the study. Follow-up visits occur every 6 years for patients aged 60–78 and every 3 years for patients aged 78+. In the case of irregular visits, the follow-up times *t* are simulated from a Weibull distribution, starting from the age of exit and continuing until the age of death or dropout: t_k ~ Weibull(shape = 5, scale = 0.4).
